# The Antioxidant Auraptene Improves Aged Oocyte Quality and Embryo Development in Mice

**DOI:** 10.3390/antiox12010087

**Published:** 2022-12-30

**Authors:** Yun-Hee Kim, Su-Yeon Lee, Eun-Young Kim, Kyeoung-Hwa Kim, Mi-Kyoung Koong, Kyung-Ah Lee

**Affiliations:** 1 Department of Biomedical Science, Institute of Reproductive Medicine, College of Life Science, CHA University, Pangyo-ro 335, Bundang-gu, Seongnam-si 13488, Republic of Korea; 2 CHA Fertility Center Daegu Station, Dalgubeol-daero 2095, Jung-gu, Daegu 41936, Republic of Korea

**Keywords:** postovulatory aging, oocyte, Auraptene, antioxidant, oxidative stress, NRF2

## Abstract

Decrease in quality of postovulatory aged oocytes occurs due to oxidative stress and leads to low fertilization and development competence. It is one of the main causes that exerting detrimental effect on the success rate in assisted reproductive technology (ART). Auraptene (AUR), a citrus coumarin, has been reported to possess an antioxidant effects in other tissues. In this study, we aimed to confirm the potential of AUR to delay the oocyte aging process by alleviating oxidative stress. Superovulated mouse oocytes in metaphase of second meiosis (MII) were exposed to 0, 1 or 10 μM AUR for 12 h of in vitro aging. AUR addition to the culture medium recovered abnormal spindle and chromosome morphology and mitigated mitochondrial distribution and mitochondrial membrane potential (ΔΨ) in aged oocytes. AUR-treated aged oocytes also showed suppressed oxidative stress, with lower reactive oxygen species (ROS) levels, higher glutathione (GSH) levels and increased expression of several genes involved in antioxidation. Furthermore, AUR significantly elevated the fertilization and embryo developmental rates. Oocytes aged with 1 μM AUR exhibited morphokinetics that were very similar to those of the control group. Altogether, these data allowed us to conclude that AUR improved the quality of aged oocytes and suggest AUR as an effective clinical supplement candidate to prevent postovulatory aging.

## 1. Introduction

Auraptene (7-geranyloxycoumarin, AUR) is a natural bioactive coumarin derivative that can be isolated from citrus fruits [1]. In a recent study, it has been shown that AUR has a variety of pharmacological actions, including antioxidative, anti-inflammatory, anticancer, neuroprotective, and immunomodulatory properties [2,3,4,5,6]. Additionally, there was no significant difference in acute and subacute toxic histopathological effects and immunotoxicity evaluation in rats [7]. With respect to controlled randomized trials, there has been no report of side effects on AUR administration in healthy adults [8] or older persons [9]. Therefore, it has been suggested to be a potential therapeutic substance in the prevention and treatment of various diseases [1]. Despite AUR’s broad range of biological activities, there is a lack of research addressing the effect of AUR in in vitro oocyte aging and embryo development.

After ovulation, female oocytes arrested at the metaphase of the second meiosis (MII) and await fertilization by spermatozoa either in the oviduct or in culture media in vitro. If fertilization does not occur over a prolonged period, oocytes progressively undergo a time-dependent deterioration in quality, which is referred to as postovulatory aging [10]. Not surprisingly, there are various oocyte defects that postovulatory aged oocytes exhibit, including spindle abnormalities, the loss of chromosomal integrity, mitochondrial dysfunction, calcium oscillation, partial cortical granule exocytosis and zona hardening [11,12,13,14]; thus, these cellular and molecular changes ultimately have damaging consequences for fertilization and subsequent embryonic development.

The mechanisms controlling postovulatory aging have not been well defined; however, there is accumulating evidence indicating that oxidative stress is strongly associated with a decline in postovulatory aged oocyte quality [15]. The aging process is accompanied by the overproduction of ROS. The shift in the balance between the production of ROS and antioxidant enzymes is disrupted, which, in turn causes oxidative stress [16]. In particular, postovulatory aging is inevitable for oocytes with increasing culture time following ovulation during assisted reproductive technologies (ARTs) which are widely used to treat infertility [17]. In addition, in vitro ART procedures contribute to the generation of oxidative stress through the technique itself and its performance in the absence of an endogenous defense system [18]. This oxidative damage may occasionally accelerate the onset of apoptosis in oocytes and ultimately influence their capacity for fertilization and embryo development. Thus, supplementation with antioxidants can be used as a safe and effective strategy to delay or prevent oocyte aging by alleviating oxidative stress.

It is essential to understand the molecular mechanism underlying how oxidative stress is relieved and antioxidant capacity is enhanced in oocyte aging. Nuclear factor erythroid-2-related factor 2 (NRF2) is a transcription factor that regulates the expression of antioxidants that protect against oxidative damage. NRF2 is maintained in the cytoplasm at a low level while undergoing constant proteasomal degradation by a negative regulator, Kelch-like ECH-associated protein 1 (KEAP1), under homeostatic conditions. In response to stress, NRF2 dissociates from KEAP1, allowing it to translocate into the nucleus and bind to DNA promoters and antioxidant response elements (AREs) [19]. Stress-inducible activation of NRF2 leads to the transcription of antioxidative genes, including NAD(P)H:quinone-oxidoreductase-1 (*Nqo1*), glutamate-cysteine ligase (*Gcl*), glutathione S-transferase (*Gst*) and glutathione peroxidase (*Gpx*). The age-dependent change in the oxidative stress response thus involves the NRF2/KEAP1 signaling system [20].

This study aimed to explore whether AUR can protect oocytes against oxidative stress during postovulatory aging and elucidate the underlying cellular and molecular mechanisms of these effects. Taken together, these data allowed us to propose the use of AUR as a candidate antioxidant culture medium supplement to improve success rates in ART programs.

## 2. Materials and Methods

### 2.1. Oocyte Collection and Culture

All animals provided by Koatech (Pyeongtack, Gyeonggi, Republic of Korea) were maintained under standard temperature and humidity conditions with a 12 h light-dark cycle at the Laboratory Animal Research Center of CHA University. Female 3-week-old ICR mice were mainly used in this study, and BDF1 mice were used for only IVF and a time-lapse imaging system. To collect MII oocytes, superovulation was induced by intraperitoneal injections of 10 IU of human chorionic gonadotropin (hCG, Sigma–Aldrich, St. Louis, MO, USA) 48 h after intraperitoneal injections of 5 IU of pregnant mares serum gonadotropin (PMSG, Daesung, Uiwang, Gyeonggi, Republic of Korea). After 16 h, the female mice were sacrificed to collect the cumulus-oocyte complex (COC) from the oviduct ampullar. To obtain cumulus-free oocytes, cumulus cells (CC) were removed by pipetting with hyaluronidase (Sigma–Aldrich). For the Young control group, oocytes were used for the experiment as soon as the cumulus cells were removed. For the aged group, denuded oocytes were washed at least three times, immediately placed in M16 medium (Sigma–Aldrich) containing 1% penicillin–streptomycin (Gibco, Grand Island, NY, USA) and cultured for 12 h at 37 °C in an atmosphere of 5% CO_2_. For the POA+AUR groups, AUR purchased from Sigma–Aldrich was dissolved in dimethyl sulfoxide (DMSO, Sigma–Aldrich) and diluted to a final concentration of 1 or 10 μM in M16 medium where the DMSO concentration did not exceed 0.01% [21]. Oocytes were cultured in M16 supplemented with different concentrations of AUR (1 or 10 μM) for 12 h at 37 °C in an atmosphere of 5% CO_2_.

### 2.2. Immunofluorescence Staining

MII oocytes were washed in phosphate-buffered saline (PBS) containing 0.1% polyvinyl alcohol (PVA), fixed in 4% paraformaldehyde (PFA) and then permeabilized with 0.2% Triton X-100 for 20 min. After 1 h of blocking in 3% bovine serum albumin (BSA) dissolved in PVA-PBS, the samples were incubated overnight at 4 °C with the primary antibody (anti-α-tubulin antibody, Santa Cruz Biotechnology, Dallas, TX, USA, 1:100). After three washes with 0.1% PVA-PBS, oocytes were labeled with secondary antibody (Alexa Fluor 488 goat anti-mouse IgG, Invitrogen, Carlsbad, CA, USA, 1:100) at room temperature (RT) for 1 h and then counterstained with 500 μM DAPI (Sigma–Aldrich) for chromosome staining. After washing with 0.1% PVA-PBS, the oocytes were mounted with a cover glass, and the fluorescence signal was measured by a confocal microscope (Leica TCS SP5 II, Leica, Wetzlar, Germany) with the same parameters.

### 2.3. JC-1 Assay for Mitochondrial Activity 

To evaluate the change in ΔΨm in the oocytes, oocytes were stained with eBioscience™ JC-1 Mitochondria Membrane Potential Dye (Thermo Fisher Scientific, Waltham, MA, USA). Oocytes were cultured in M16 medium supplemented with JC-1 (1 μg/mL) for 30 min at 37 °C in 5% CO_2_ and then washed with PVA-PBS. The oocytes were mounted with mineral oil, and signals in the red and green fluorescence channels were immediately captured under a confocal microscope. For quantitative analysis, LAS AF Lite software (version 2.3.5 build 5379) was used to measure the signal intensities, and ΔΨm was calculated as the ratio of the red and green signals. In the case of a higher ΔΨm, the ratio was shifted more toward red fluorescence, and the color of the emitted fluorescence changed from green to red. Conversely, with a lower ΔΨm, the ratio decreased and green fluorescence was exhibited. Therefore, healthy mitochondria show a higher red to green fluorescence intensity ratio in a state with a high ΔΨm.

### 2.4. Measurement of Intracellular ROS and Glutathione (GSH) Levels

An OxiSelect™ Intracellular ROS Assay kit (STA-342, Cell Biolabs, San Diego, CA, USA) and CellTracker™ Blue CMF2HC dye (C12881, Invitrogen) were used to measure the intracellular ROS and GSH levels in denuded oocytes. In brief, oocytes were incubated in M16 medium supplemented with 1X 2,7-dichlorodihydrofluorescein diacetate (DCFH-DA) for 30 min at 37 °C or 0.1% PVA-PBS containing 1 μg/mL CMF2HC in the dark at RT. After washing, the fluorescence signal was measured by a confocal microscope with the same parameters. The intensity of the ROS and GSH signals in each oocyte from different groups was measured with ImageJ software (NIMH, Bethesda, MD, USA).

### 2.5. Real-Time RT-PCR

To detect NRF2 signaling-related transcript expression, mRNA was isolated from 20 MII oocytes in group using the Dynabeads mRNA DIRECT kit (Invitrogen) as described in a previous report [22]. Magnetic beads covalently bound to oligo-dT primer were specifically targeted and captured by base pairing poly(A) tail residues at the mRNA 3′ end. After washing with buffers, RNA was eluted and reverse-transcribed into complementary DNA (cDNA) using M-MLV-RT (Promega, Madison, WI, USA) with oligo(dT) primer. The synthesized cDNA was stored at −20 °C until use. The sequences of the gene-specific primers used for real-time RT–PCR are listed in Table 1. The expression of the transcripts was quantified using iQ SYBR Green Supermix (Bio-Rad, Hercules, CA, USA) with a CFX96 Touch Real-Time PCR Detection System (Bio-Rad). Relative gene expression levels were calculated using the 2^−∆∆Ct^ method and analyzed.

### 2.6. Western Blot

Lysates from 300 mouse oocytes per sample were prepared in RIPA buffer containing 100X phosphatase inhibitor. The samples were boiled for 5 min at 95 °C and loaded onto 8% SDS–PAGE, and the protein was separated using electrophoresis and transferred from the gel to PVDF membranes. The membranes were blocked and incubated with anti-NRF2 antibody (ab92946, Abcam, Cambridge, UK) and anti-α-tubulin antibody (sc-8035, Santa Cruz Biotechnology, Dallas, TX, USA) diluted 1:1000 in blocking solution at 4 °C overnight. Next, HRP-conjugated anti-rabbit secondary antibody or HRP-conjugated anti-mouse secondary antibody diluted 1:5000 in blocking solution was added for 1 h of incubation at RT. After washing with TBST, an enhanced chemiluminescence kit was used to detect the specific proteins. The bands were visualized using the ChemiDoc XRS+ system (Bio-Rad). The relative intensity of each band was analyzed using Image Lab software (version 6.1.0 build 7, Standard Edition, Bio-Rad).

### 2.7. In Vitro Fertilization

The cauda epididymis of 8- to 12-week-old BDF-1 male mice was slightly incised, and sperm were squeezed out using a needle. Sperm were placed into a dish of human tubal fluid (HTF, Sigma–Aldrich) medium covered with mineral oil and then incubated for 1 h at 37 °C in 5% CO_2_ to allow the sperm to capacitate. At the end of 12 h aging time of POA groups, young oocytes were collected and the CC removed, and the Young group was performed IVF simultaneously with the POA oocyte group. MII oocytes of all groups were added to the HTF droplets at a concentration of 4 × 10^5^ sperm per 1 mL for 6 h at 37 °C in 5% CO_2_. To remove the sperm, the oocytes were repeatedly and thoroughly washed and transferred to plain M16 medium containing 0.1 mM EDTA that had been equilibrated overnight in the incubator for 120 h of culture.

### 2.8. Time-Lapse Imaging System

A time-lapse imaging system was used to track phenotypic changes and the speed of embryo development during in vitro culture. Embryos were cultured under a time-lapse microscope (iEM900, CNC Biotech, Seoul, Republic of Korea), and their developmental events were examined and evaluated. A culture dish containing two pronuclei (2PN) zygotes was placed in the microscope. Images were automatically captured every 5 min for 120 h, and the sequential time-lapse images were converted into movie files. The morphokinetics of the embryos were recorded according to the timing at which specific events occurred: t0, time to insemination; tf, pronuclei fading and the entry into the first embryonic M-phase; t2, t3, t4, t5, t6, t7, and t8: time in hours post insemination (HPI) required for the embryos to reach the 2-, 3-, 4-, 5-, 6-, 7-, and 8-cell stage, respectively; tErB, time to start formation of the blastocoel cavity; tBL: half or more of the blastocoel cavity formed; CC, length of cell cycle; and S, synchronicity or the round of cleavage division.

### 2.9. Statistical Analysis

Statistical analysis was performed using IBM SPSS Statistics 22.0 software (IBM Corp., Armonk, NY, USA). Data are presented as the mean ± standard error of the mean (SEM). The mean and SEM of the data were calculated and statistically analyzed by analysis of variance (ANOVA) followed by post hoc analysis and Student’s t test. Differences were considered biologically significant at *p* values < 0.05.

## 3. Results

### 3.1. AUR Maintains Spindle Assembly and Chromosome Alignment during Postovulatory Aging

**Figure 1:**
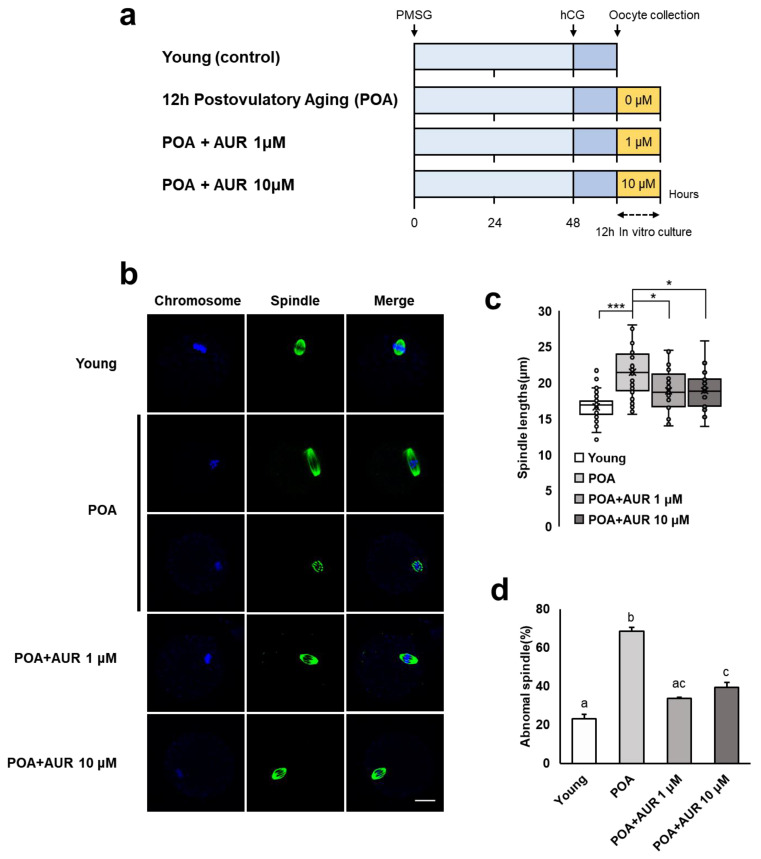
AUR protected the organizational dynamics of spindles and chromosomes during postovulatory aging. (**a**) A schematic diagram to illustrate the experimental design to investigate how AUR impact postovulatory aged oocytes. (**b**) Representative images of meiotic spindles and chromosomes in young and aged oocytes with or without AUR administration. Spindles were stained with α-tubulin (a spindle marker, green), and chromosomes were stained with DAPI (DNA, blue); scale bar = 20 μm. (**c**) Lengths of the spindles in each group of oocytes. Dots of boxplot show individual values of each observation. (**d**) Percentages of abnormal spindles in oocytes in each group. Data are presented as the means ± SEMs of at least three independent experiments. Significant difference indicated by lowercase letters of the alphabet, *p* < 0.05, * *p* < 0.05, *** *p* < 0.001.

To investigate whether AUR has beneficial effects on aging oocytes, oocytes were additionally cultured in vitro for 12 h to induce postovulatory aging in the presence of 0, 1, or 10 μM AUR in the culture medium (Figure 1a). We examined spindle organization and chromosome alignment in aged oocytes. As shown in Figure 1b, young control MII oocytes displayed a barrel-shaped spindle with chromosomes that were well aligned at the equatorial plate. In contrast, in aged oocytes without AUR treatment, the number of elongated spindles or disrupted spindles with misaligned chromosomes was markedly increased compared to that in young oocytes (spindle length: Figure 1c; abnormal rate: Figure 1d). However, the AUR-treated aged oocytes showed a significantly reduced number of abnormal spindles compared to the nontreated aged oocytes. The above results showed that AUR can suppress abnormalities in spindle organization and chromosome alignment in in vitro aged oocytes.

### 3.2. AUR Recovers Mitochondrial Dysfunction in Postovulatory Aged Oocytes

**Figure 2:**
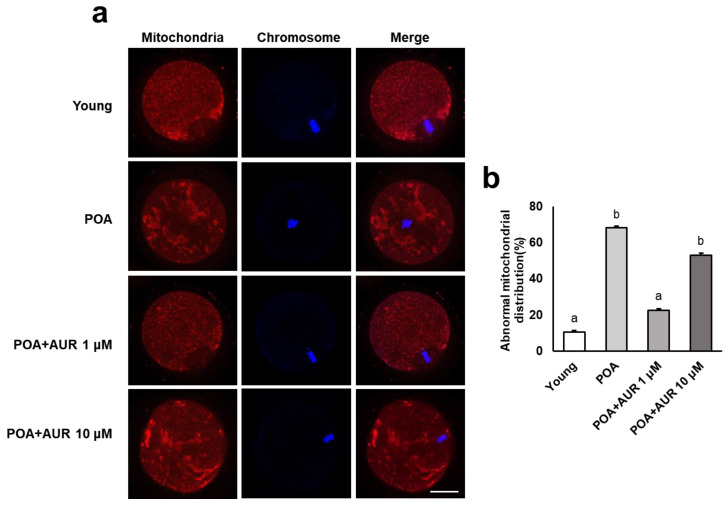
AUR affected mitochondrial distribution during postovulatory aging. (**a**) Representative images of mitochondrial distribution in young and aged oocytes with or without AUR administration. Oocytes were stained with MitoTracker (mitochondria marker, red), and chromosomes were stained with DAPI (DNA, blue); scale bar = 100 μm. (**b**) Percentages of abnormal mitochondrial distribution in each group of oocytes. Data are presented as the means ± SEMs of at least three independent experiments. Significant difference indicated by lowercase letters of the alphabet, *p* < 0.001.

Mitochondrial dynamics is an important source of ATP production within both oocytes and embryos and is closely related to oxidative stress [23]. Therefore, we tested the activity and function of mitochondria following postovulatory aging by evaluating the distribution pattern and ΔΨm. Normal oocytes showed even dispersion throughout the cytoplasm and accumulated around the chromosomes, while oocytes aged in vitro showed an uneven distribution with large cluster aggregates in the cytoplasm (Figure 2a). These abnormal distribution patterns were significantly reduced in the 1 μM AUR-treated group but not in the 10 μM AUR group (Figure 2b).

**Figure 3:**
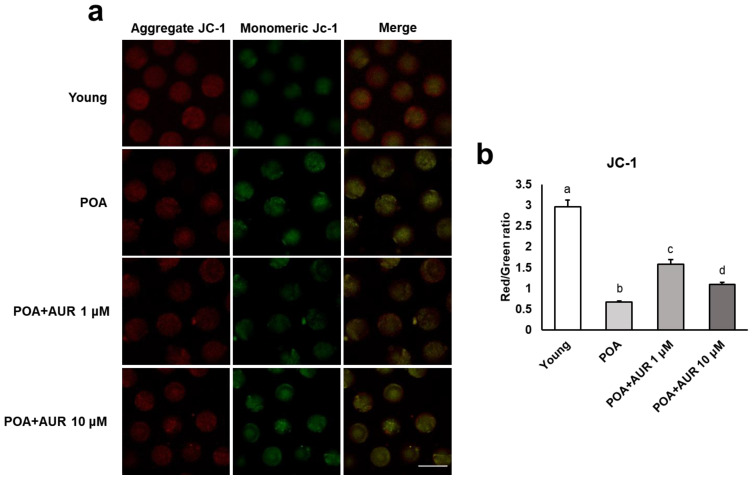
AUR enhanced mitochondrial function by raising ΔΨm during postovulatory aging. (**a**) Representative images of mitochondrial ΔΨm in young and aged oocytes with or without AUR administration. Oocytes were stained with the cationic dye JC-1, which can selectively enter mitochondria and reversibly turn the emitted green fluorescence into red depending upon the mitochondrial membrane potential (aggregated JC-1: high potential marker, red; monomeric JC-1: low potential marker, green); scale bar = 20 μm. (**b**) The ratio of red to green fluorescence intensity in each group of oocytes. Data are presented as the means ± SEMs of at least three independent experiments. Significant difference indicated by lowercase letters of the alphabet, *p* < 0.001.

When the cationic fluorescent dye JC-1 encounters a high membrane potential, it accumulates inside the mitochondria, aggregates in a concentration-dependent manner and appears red. In contrast, at low membrane potential, JC-1 is present as a monomer and appears green (Figure 3a). Quantitative analysis revealed that the ratio of red to green fluorescence significantly decreased in aged oocytes but this ratio was rescued, i.e., increased, in AUR-treated oocytes (Figure 3b). These results indicate that AUR administration during postovulatory aging can help oocytes maintain normal mitochondrial activity and function.

### 3.3. AUR Decreases the Intracellular ROS Level and Increases the GSH Level in Aged Oocytes

**Figure 4:**
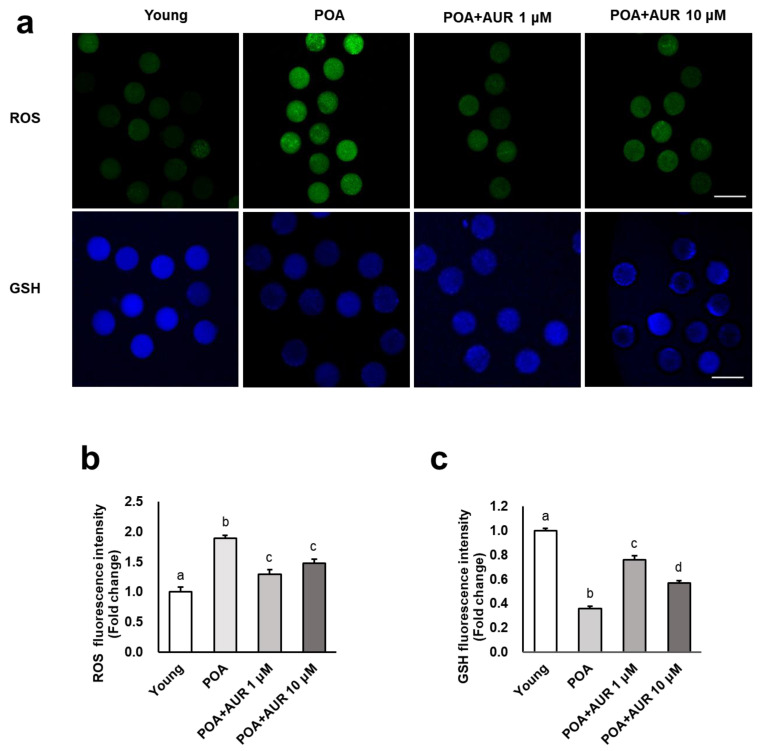
AUR decreased intracellular ROS and elevated GSH during postovulatory aging. (**a**) Representative images of reactive oxygen species (ROS) and glutathione (GSH) distribution in the oocytes in the Young, POA, POA+AUR 1 μM and POA+AUR 10 μM groups. Oocytes were stained with DCFH-DA (green) or CMF2HC (blue), scale bar = 20 μm. (**b**) Fluorescence intensities denoting intracellular ROS distribution in oocytes in all groups. (**c**) Fluorescence intensities of intracellular GSH staining distribution in oocytes in all groups. Data are presented as the means ± SEMs of at least three independent experiments. Significant difference indicated by lowercase letters of the alphabet, *p* < 0.01.

Oxidative stress has been considered a key mechanism underlying cellular aging [15]. To evaluate the potential of AUR to protect oocytes from oxidative stress during postovulatory aging, we measured the levels of intracellular ROS in postovulatory aged oocytes with DCFH-DA. As shown in Figure 4a,b, the ROS levels in aged oocytes were significantly higher than those in young oocytes, while aged oocytes in the presence of AUR had significantly reduced ROS levels. GSH is an important intracellular antioxidant that eliminates ROS in oocytes [24], so the level of GSH was detected by CMF2HC dye. 

The intracellular GSH level in aged oocytes was significantly lower than that in young oocytes, but the GSH content in the aged groups treated with AUR was significantly higher than that in the untreated aged group (Figure 4a,c). These observations suggested that AUR is able to reverse the oxidative damage caused by ROS accumulation and the lack of GSH during postovulatory aging.

### 3.4. AUR Reduces Oxidative Stress by Regulating the NRF2 Pathway in Aged Oocytes

**Figure 5:**
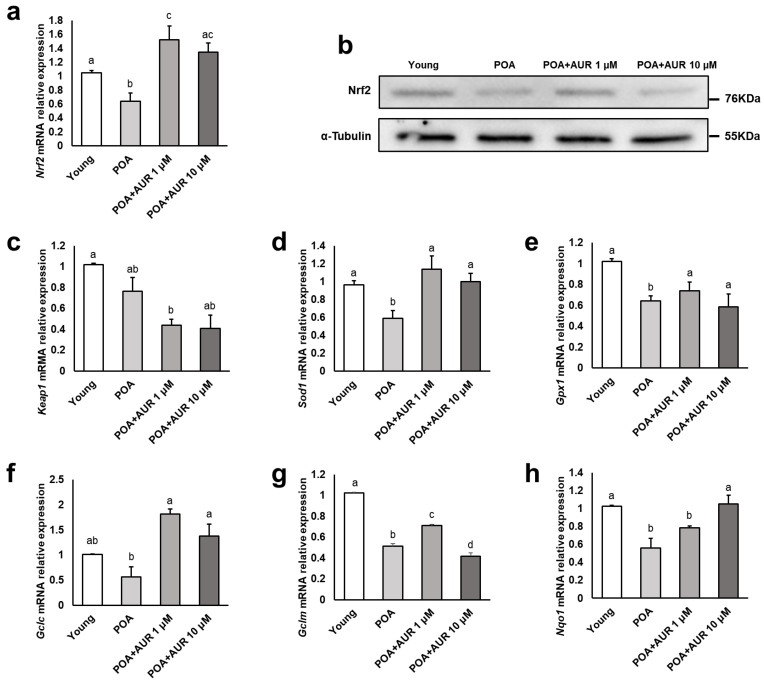
AUR upregulated the expression of NRF2-related genes during postovulatory aging. (**a**,**b**) Expression patterns of NRF2 mRNA and protein in postovulatory aged oocytes. (**c**–**h**) Transcription levels of NRF2-related genes (*Keap1*, *Gclc*, *Gclm*, *Gpx1*, *Sod1* and *Nqo1*), *p* < 0.05. Data are presented as the means ± SEMs of at least three independent experiments. Significant difference indicated by lowercase letters of the alphabet, *p* < 0.05.

In vitro postovulatory aging is accompanied by excessive ROS formation, and antioxidant deficiency may lead to oxidative stress. Given the important role of NRF2 as a key regulator of the antioxidant defense system against oxidative stress [20], we examined the relative mRNA expression of *Nrf2* and its target genes that are related to the antioxidant defense system in oocytes. Notably, the expression of NRF2 at the transcript and protein levels in the aged group was significantly decreased compared with that in the young group, but the AUR-treated aged group displayed significantly increased levels (Figure 5a,b). However, the transcript level of *Keap1*, which represses *Nrf2* gene transcription, did not differ between the young and aged groups (Figure 5c). When examining the expression of genes involved in primary antioxidant cellular defense, the transcripts of *Gclm*, *Gpx1*, *Sod1* and *Nqo1* were significantly decreased in the aged group compared with the young group, and the AUR-treated aged group had significantly increased mRNA levels of these genes, with the exception of *Nqo1* (Figure 5d–h). These results suggest that the generation of oxidative stress during postovulatory aging is relieved by activating antioxidative effects through the NRF2 signaling pathway under the effect of AUR.

### 3.5. AUR Improves the Fertilization and Preimplantation Embryo Development Potential of Postovulatory Aged Oocytes

**Figure 6:**
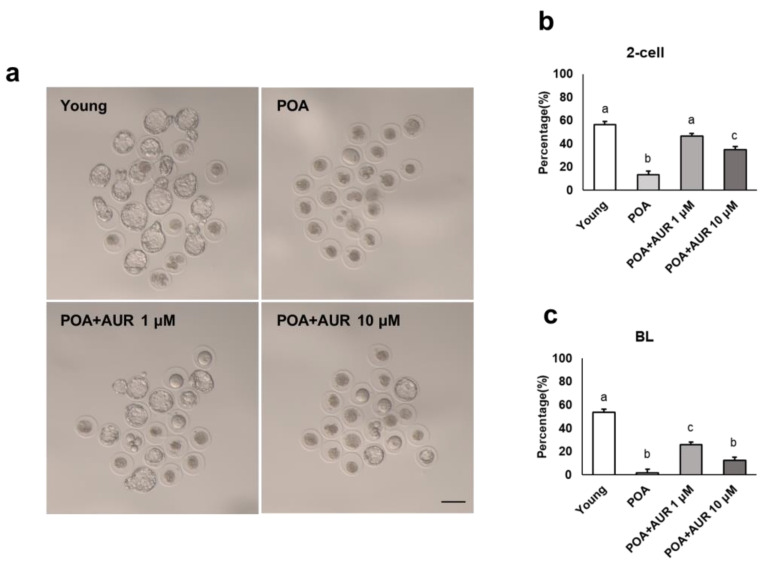
AUR promoted the fertilization and preimplantation development of postovulatory aged oocytes. (**a**) Representative images of blastocysts from young, aged and AUR-treated aged oocytes. Scale bar = 100 μm. (**b**,**c**) Quantitative analysis of embryo 2-cell and blastocyst formation rates. Data are presented as the means ± SEMs of at least three independent experiments. Significant difference indicated by lowercase letters of the alphabet, *p* < 0.01.

Above, we confirmed that AUR improves the quality of aged oocytes; therefore, we also tried to determine whether AUR affects fertilization and embryo development. As shown in Figure 6a–c, in the young group, 57.8% of the oocytes could develop into 2-cell (2C) embryos, and 55% could develop to the blastocyst stage. For 12 h aged oocytes, the proportions of 2C and blastocyst embryos decreased to 13.6% and 1.6%, respectively. However, in the AUR 1 μM- or 10 μM-supplemented aged oocyte group, the proportions of 2C embryos (47.6% and 35%, respectively) and blastocyst formation (26% and 12.5%, respectively) were significantly increased (Figure 6a–c).

**Figure 7:**
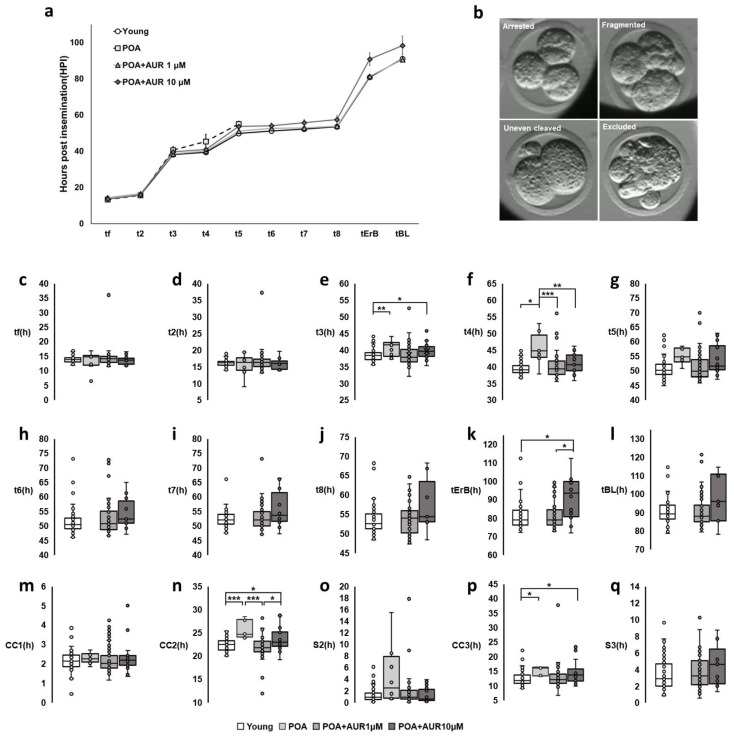
AUR advanced the morphokinetics of embryos from postovulatory aged oocytes. (**a**) Comparison of the morphokinetics of embryos derived from oocytes of the Young, POA, POA+AUR 1 μM and POA+AUR 10 μM groups. (**b**) Representative images of embryos from aged oocytes that had not reached the blastocyst stage. (**c**–**q**) t2, t3, t4, t5, t6, t7, and t8: time in hours post insemination (HPI) required for embryos to reach the 2-, 3-, 4-, 5-, 6-, 7-, and 8-cell stage, respectively; tErB: time to start formation of blastocoel cavity; tBL: half or more of blastocoel cavity had formed; CC: length of the cell cycle; S: synchronicity or round of cleavage division. Dots of boxplot show individual values of each observation. Data are presented as the means ± SEMs of at least three independent experiments, * *p* < 0.05, ** *p* < 0.01, *** *p* < 0.001.

Additionally, a time-lapse imaging system was used to assess the morphology and timing of successive divisions, compaction and blastocoel formation during embryo development. The results of the time-lapse imaging system were analyzed, with fertilized embryos showing 2PN. Therefore, the number of *n* in each group was different depending on the fertilization rate (*n* = 65, 12, 60, 25 embryos in each group). In the case of aged oocytes, the fertilization rate was low, and the number of embryos was small. In embryos derived from aged oocytes, the cleavage time from t3 to t5, CC2 (t3-t2) and CC3 (t5-t3) were significantly increased compared to embryos from young oocytes (Figure 7c–q) and resulted in failure to develop to the blastocyst stage with an increased incidence of unequal-sized blastomeres, developmental arrest and fragmentation (Figure 7a,b, Video S1).

The morphokinetic parameters of the embryos from aged oocytes with 1 μM AUR treatment were comparable to those of embryos from young oocytes with no statistically significant difference (Figure 7c–q, Video S1). However, the embryos from aged oocytes with 10 μM AUR were able to form a blastocyst, but the cleavage time was longer than that of the embryos from young oocytes. Additionally, these blastocysts mostly underwent cell exclusion during compaction, excluding cell fragments not participating in blastocyst formation [25], and did not develop to a sufficient size for subsequent expansion or hatching (Figure 7a,b, Video S2). Collectively, these results suggest that 1 μM AUR can delay postovulatory aging and improve the fertilization and early embryonic development of aging oocytes.

## 4. Discussion

Postovulatory aging in vitro is an important detrimental cause of failure in ART procedures. The age-related decline in oocyte quality compromises subsequent fertilization and embryo development. Therefore, in the current study, we investigated whether the antioxidant AUR can effectively improve the quality of aged oocytes to increase the fertilization success rate and further embryo development. We applied particular focus to relieving oxidative stress as a major contributor to the aging of oocytes. AUR, a well-known coumarin is well known as an outstanding antioxidant with radical scavenger activities. Antioxidative activity of AUR is higher than reference substances (Ascorbic acid and Trolox^®^) [4]. AUR with antioxidant activities has been acknowledged in several studies using other experimental models [26]. The anti-inflammatory and antioxidative effects of AUR also increased oocyte maturation and the fertilization rates of oocytes in an in vivo polycystic ovarian syndrome (PCOS) model [27]. AUR inhibited ROS generation and enhanced mitochondrial respiration through induction of NRF2 in a Parkinson’s disease mouse model [28] and activated antioxidant enzymes and the mitochondrial unfolded protein response, which improves junction assembly in cerebrovascular endothelial cells [29]. AUR also induced hepatic GST and NQO1 by activating the antioxidant response in *Nrf2* knockout mice [30]. Moreover, Hassanein and colleagues explained the pharmacological and therapeutic effects of coumarins related to antioxidation in the KEAP1-NRF2-ARE signaling pathway [31]. Furthermore, *Nrf2* deletion was associated with a wide variety of pathologies, including aging and age-related diseases [20,32]. NRF2 localizes around the meiotic spindle and is associated with the proper formation or stabilization of the spindle, and its expression is significantly decreased in aged oocytes, resulting in the loss of GSH synthesis [33]. In addition, accumulating studies have indicated that the activation of NRF2 signaling could be a potential regulator to suppress oxidative stress to normalize reproductive functions [34,35].

Thus, we hypothesized that supplementing culture media with the antioxidant AUR might prevent postovulatory aging by suppressing oxidative stress through the modulation pf NRF2 signaling. AUR has never been tested before in in vitro oocyte culture, so we used concentrations of 1 μM and 10 μM in the culture medium with different cell lines to show its pharmacological effects [36,37]. We first assessed the meiotic spindle assembly and chromosome alignment of postovulatory aged oocytes with or without AUR. Meiotic chromosomes contain the oocyte’s genetic information, and an intact meiotic spindle is responsible for the correct segregation of chromosomes. Additionally, it was reported that 12 h aged oocytes interfered with the proper involvement of the spindle assembly checkpoint (SAC) transcripts and functional proteins and exhibited aneuploidy, which occurs in aged oocytes or early embryonic developmental stages at the same frequency [38]. In line with previous observations, we confirmed that the spindle changes abnormally during oocyte aging, but we found that aged oocytes retain normal spindle and chromosome structures after AUR supplementation. In addition, we also analyzed mitochondrial activity and functions in aged oocytes. Oocyte mitochondria are the organelles that generate ATP via oxidative phosphorylation, contribute to biosynthesis, and control redox homeostasis during oocyte maturation, fertilization, and embryo development. These mitochondrial activities must be supported in mature oocytes and embryos [39]. Previous studies have shown that changes in the distribution and function of mitochondria are associated with aging [40]. Our data demonstrated that AUR protects the function of the mitochondria by rescuing mitochondrial distribution patterns and increasing the membrane potential. Therefore, we determined that AUR could improve oocyte quality during postovulatory aging in vitro.

As a major cause of oxidative stress, aged oocytes exhibit susceptibility to raised ROS levels and concomitantly decreased antioxidant protection. GSH is the most plentiful antioxidant in cells and impacts cytoplasmic maturation, correlating with oocyte fertilization and subsequent development [41]. It has also been reported that the level of GSH is lower in postovulatory aged mouse oocytes [17]. Consistent with previous studies, we observed that AUR significantly diminished the levels of intracellular ROS while increasing GSH contents within in vitro aged oocytes, indicating that AUR alleviates oxidative stress in aged oocytes. Additionally, to track the mechanism of the antioxidative activities of AUR, we detected the expression of NRF2 and its downstream antioxidant enzymes. Indeed, the NRF2 pathway plays a significant role in activating the response to oxidative stress. ROS scavenging enzymes such as SOD1 and GPx1 are front-line mediators of redox equilibrium [42]. SOD1 is associated with the suppression of segregation defects during aging in oocytes [43], and GPx reinforces oocyte and embryo developmental capacity [44]. In addition, knockout of *Gclm*, the regulatory subunit that constitutes the GCL complex, which is the rate-limiting step in the synthesis of GSH, causes litter size reduction by damaging embryo viability during early embryonic development [45]. Thus, increasing the expression of these antioxidant enzymes is important to protect oocytes and embryos from oxidative stress brought about by aging. In our study, we confirmed that the expression levels of *Nrf2* and antioxidant enzyme genes (*Sod1*, *Gpx1*, *Gclc*, *Gclm*, and *Nqo1*) decreased under in vitro aging conditions and but increased after AUR treatment. These results indicate the potential mechanisms of the anti-aging effects of AUR through NRF2-mediated antioxidant signaling pathways, effectively removing ROS in postovulatory aged oocytes.

Decreases in the fertilization and preimplantation embryo development rates can be attributed to multiple biochemical and functional alterations to the oocyte that accumulate with postovulatory aging [14]. We investigated the effect of AUR on the fertilization and embryonic development rates of in vitro postovulatory aged oocytes and evaluated the morphokinetic parameters of preimplantation embryos using time-lapse imaging. Continuous noninvasive monitoring of embryo development allows the selection of embryos with the best implantation potential for ART [46]. Moreover, the morphokinetic parameters provide sensitive indicator of quality of embryos culture in vitro [47]. In this study, the fertilization rate of the aged oocytes was greatly reduced, and even after fertilization, the timing of cleavage was delayed, and development to the blastocyst stage rarely occurred. Embryos from aged oocytes degenerated by showing several morphologies, including arrest, irregular divisions, and fragment formation. These factors also negatively correlate with embryo development and implantation [48]. In aged oocytes treated with both concentrations of AUR, the fertilization rate and embryonic development rate were higher than those in aged oocytes. However, this situation was different depending on the concentration of AUR. In aged oocytes administered 10 μM AUR, the embryos formed blastocysts, unlike aged oocytes without AUR, but development took longer, particularly for tErB. It seems likely that cell exclusion occurred at the cavitation of morula stage embryos or extrusion at the postcompaction stage, so the embryo did not grow to a full size that was large enough to expand or escape from the zona pellucida to hatch. Cell exclusion during compaction (morula stage) or later at the blastulation stage is an attempt to self-correct by putting out cells that may be mosaic. However, embryos that exclude cells early in development would have higher aneuploidy than late exclusion embryos [49]. On the other hand, with 1 μM AUR, embryo development was relatively shorter and not different from that in the young oocyte group. Therefore, these results indicated that 1 μM AUR has the potential to improve the quality of aged oocytes after ovulation, the fertilization rate and embryonic development.

ART is the only way to effectively solve the problems of infertile patients that are produced by a variety of factors. Nevertheless, to reduce the risk of ART failure, there is a need for a series of clinical trials involving the development of a strategy to gain time to protect gametes and embryos from oxidative stress. Attempts have been made to minimize oxidative stress by supplementation with antioxidants. Factually, supplementation with natural antioxidants such as melatonin, coenzyme Q10 (CoQ10), resveratrol, vitamins, etc., has already been reported in in vitro and in vivo studies using animal models as well as human clinical studies. Following is a brief summary of the characteristics of some of these antioxidants. First, melatonin improves oocyte quality through the direct scavenging of ROS and indirect action-mediated receptors [50]. The concentrations of melatonin increased in ovarian follicular fluid and oocytes to reduce oxidative stress in infertile patients after in vivo administration [51]. The clinical application of melatonin has been shown to have a good influence on human oocyte maturation, fertilization and embryo development rates [52]. On the other hand, melatonin showed no significant impact on the number of MII oocytes, clinical pregnancy rate, or numbers of live births or miscarriages [53]. CoQ10, an electron carrier in the mitochondrial respiration chain, improves the quality of oocytes by reducing oxidative stress and enhancing mitochondrial function in animal models [54]. In human clinical studies, oral CoQ10 supplementation increased the number of mature follicles and established a more appropriate environment for follicle development [55,56]. However, these factors bring about no significant differences in pregnancy, miscarriage, or live birth rates [57]. Rather, during in vitro maturation, the addition of either CoQ10 or MitoQ (a CoQ10 analog) to culture media can increase maturation rates and improve the quality of human oocytes by reducing aneuploidy rates [58] or decreasing chromosomal defects [59], respectively. Resveratrol has been studied for its beneficial effects on oocyte maturation and embryo development in experimental models [60,61]. However, there is a dearth of information from human IVF data. Bahramrezaie et al. [62] conducted a randomized controlled trial in which sixty-two infertile PCOS patients received 800 mg/day resveratrol orally for 40 days. As a result, the high-quality oocyte and embryo rates were higher in the resveratrol supplementation group and the expression of the VEGF and HIF1 genes was reduced in granulosa cells through the angiogenesis pathway of granulosa cells [62]. Nevertheless, it is understood that resveratrol also inhibits decidualization in the uterine endometrium, so its intake should be avoided after ovulation and during pregnancy [63]. There is also limited clinical information regarding vitamins. Several studies have investigated whether vitamins play an important role as antioxidants by evaluating the correlation between the levels of vitamins in the serum or follicular fluid and IVF outcomes [64,65]. 

Similarly, although it is still difficult to validate the outcomes obtained using animal models, the development of therapies and clinical applications using safe and promising antioxidants is important and should be continuously required in the reproductive field. Here, our findings demonstrated that AUR may assist as another antioxidant supplements to the culture media of oocyte for improving oocyte and embryo quality. This suggests that treating oocytes with AUR in the culture media can allow more time for successful fertilization and embryo development. 

## 5. Conclusions

**Figure 8:**
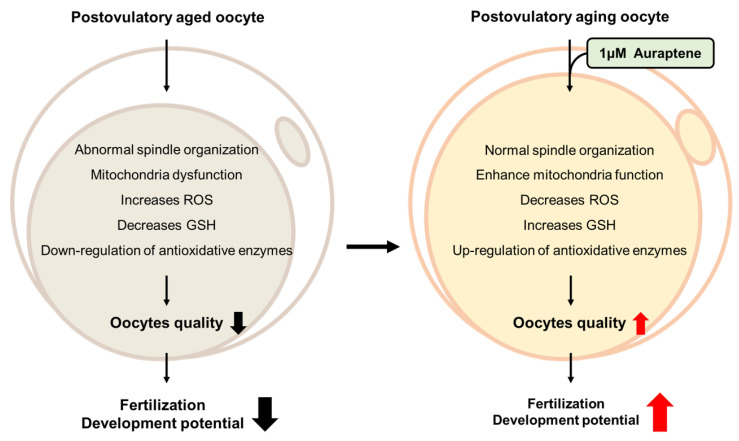
Auraptene can effectively delay postovulatory oocyte aging in vitro by alleviating oxidative stress. Briefly, we found that AUR inhibited not only spindle defects but also mitochondrial dysfunctions. In addition, the AUR treatment showed exhibit high GSH level and increase expression of antioxidant enzymes (*Nrf2*, *Gclm*, *Gclc*, *Gpx1* and *Sod1*) while decreasing the ROS level. Moreover, AUR can improve fertilization and preimplantation embryo development. Thus, we conclude that AUR may be able to provide as a potent antioxidant in clinical.

As the time that ovulated oocytes are exposed to an in vitro environment without sperm activation increases, the quality of the ovulated oocytes decreases through the aging process. AUR may be resolved the oocyte aging by providing safe interventional strategies with natural antioxidative compound. In the present study, we confirmed that AUR had positive effects on postovulatory aged oocytes, diminished free radicals and activated the antioxidant system controlling the NRF2 pathway, thereby moderating oxidative stress and consequently improving fertilization and embryonic development (Figure 8). Therefore, we propose that AUR could be a novel and effective candidate for delaying the aging process in oocytes, and in particular, the utility of antioxidants during ART techniques.

## Figures and Tables

**Table 1:** Primers used for real-time RT–PCR.

Genes	Accession No.	Forward	Reverse	Product Size (bp)
*Nrf2*	NM_010902.4	TGGAGAACATTGTCGAGCTG	TGCTTTTGGGAACAAGGAAC	237
*Keap1*	NM_001110307.1	GGCAGGACCAGTTGAACAGT	ATCACTGTCCGGGTCATAGC	188
*Sod1*	NM_011434.2	TGCTTTTGGGAACAAGGAAC	CACCTTTGCCCAAGTCATCT	216
*Cat*	NM_009804.2	CCTGACATGGTCTGGGACTT	CAAGTTTTTGATGCCCTGGT	201
*Nqo1*	NM_008706.5	CAGATCCTGGAAGGATGGAA	TCTGGTTGTCAGCTGGAATG	202
*Gpx1*	NM_008160.6	GTCCACCGTGTATGCCTTCT	TCTGCAGATCGTTCATCTCG	152
*Gclc*	NM_010295.2	CAATGGGAAGGAAGGGGTAT	TCAGGATGGTTTGCAATGAA	186
*Gclm*	NM_008129.4	TGGAGCAGCTGTATCAGTGG	AGAGCAGTTCTTTCGGGTCA	150
*Gapdh*	NM_001289726.1	ACCACAGTCCATGCCATCAC	TCCACCACCCTGTTGCTGTA	171
*H1foo*	NM_001346702.1	TCCACCACAAGTACCCGACA	GGCACAGGCTTTCTTTCTCT	173

## Data Availability

All the data is contained within the article.

## Supplementary Materials

The following supporting information can be downloaded at https://www.mdpi.com/article/10.3390/antiox12010087/s1: Video S1: Comparison of embryo development, Video S2: Cell exclusion.
